# A DEAD-box protein regulates ribosome assembly through control of ribosomal protein synthesis

**DOI:** 10.1093/nar/gkz502

**Published:** 2019-06-12

**Authors:** Isabelle Iost, Chaitanya Jain

**Affiliations:** 1 ARNA Laboratory, INSERM U1212, CNRS UMR 5320, Université de Bordeaux, France; 2 Department of Biochemistry and Molecular Biology, University of Miami Miller School of Medicine, Miami, FL 33136, USA

## Abstract

DEAD-box proteins (DBPs) comprise a large family of proteins that most commonly have been identified as regulators of ribosome assembly. The *Escherichia coli* DBP, SrmB, represents a model bacterial DBP whose absence impairs formation of the large ribosomal subunit (LSU). To define the basis for SrmB function, suppressors of the ribosomal defect of Δ*srmB* strains were isolated. The major class of suppressors was found to map to the 5′ untranslated region (UTR) of the *rplM-rpsI* operon, which encodes the ribosomal proteins (r-proteins) L13 and S9. An analysis of protein abundance indicated that both r-proteins are under-produced in the Δ*srmB* strain, but are increased in these suppressors, implicating r-protein underproduction as the molecular basis for the observed ribosomal defects. Reduced r-protein synthesis was determined to be caused by intrinsic transcription termination within the *rplM* 5′ UTR that is abrogated by SrmB. These results reveal a specific mechanism for DBP regulation of ribosomal assembly, indirectly mediated through its effects on r-protein expression.

## INTRODUCTION

DEAD-box proteins (DBPs) comprise a large family of highly conserved RNA-remodeling factors that are characterized by an *in vitro* ATP-dependent RNA helicase activity (1–3). The characteristic features of DBPs are 13 conserved amino-acid motifs, including the eponymous DEAD motif, distributed over two RecA-like structural domains that encompass 300–400 amino-acids. Biochemical and structural studies on DBPs have yielded important insights into the mechanism of RNA duplex unwinding, including a key role for ATP in this process (4,5). In particular, ATP binding to form a DBP–RNA–ATP ternary complex results in a conformational change that kinks the RNA, resulting in local strand separation (6). In additional, some DBPs also promote ATP-independent reactions such as RNA–RNA strand annealing, RNA strand exchange and RNA chaperone activity *in vitro*, but the physiological relevance of such activities is unclear (7).

DBPs have been implicated in a variety of RNA-related processes, including translation, transcription, splicing, RNA export and ribosome assembly; but arguably, the most common function of these proteins is in ribosome assembly (3). In *Saccharomyces cerevisiae*, at least 15 out of the 26 DBPs are involved in ribosome assembly, of which 12 are essential for viability. Similarly, four of the five *E. coli* DBPs and nearly half of the 37 human DBPs are implicated in ribosome assembly (8). Different mechanisms have been proposed to explain the requirement of DBPs for efficient ribosome assembly. One role, exemplified, by the *S. cerevisiae* DBP Dbp4 and the human DDX21 proteins, involves the remodeling of pre-rRNAs to enable the association or dissociation of small nuclear RNAs (snoRNAs), the guide RNAs that direct methylation or pseudouridylation of specific rRNA bases (9,10). Other DBPs, such as Dbp3 promote rRNA processing, possibly by remodeling structured RNA to increase the accessibility of processing RNases (11). Yet other proposed mechanisms invoke the rRNA remodeling function of DBPs to promote the acquisition of on-pathway conformations or to resolve kinetically trapped structures. Each of these mechanisms is based on direct and specific interactions between DBPs and rRNA.

Like many other DBPs, the *Escherichia coli* DBP, SrmB, is implicated in ribosome assembly (12,13). Prior studies showed that Δ*srmB* strains grow as well as wild-type (wt) strains at physiological temperatures, but display a pronounced growth and ribosome assembly defect at low temperatures, similar to the phenotype exhibited by r-proteins or rRNA mutants (13,14). Under these conditions, Δ*srmB* strains exhibit a substantial accumulation of 40S ribosomal particles comprised of incompletely formed LSUs. In particular, these particles lack L13, one of the five r-proteins essential for early assembly *in vitro*, implicating this DBP at an early stage of LSU assembly. In addition, by using a biochemical approach, it was shown that SrmB is tethered to the nascent large subunit through interactions with r-proteins L4 and L24 (15). Furthermore, mutations in 23S and 5S rRNAs that can suppress the Δ*srmB* growth defect were identified, suggesting that SrmB may assist the folding of these rRNA regions (16). However, the growth-defect suppression was only partial, suggesting that SrmB plays other roles in ribosome assembly. Thus, SrmB represents a model protein to investigate DBP function in ribosome assembly.

To gain additional insights into the function of SrmB in ribosome assembly, we used a genetic approach to identify suppressors of the cold-sensitive (*cs*) growth defect of Δ*srmB* strains. Through the identification and characterization of these suppressors, we show that a key role of SrmB is fundamentally different from the mechanisms that have been previously proposed to define DBP function in ribosome assembly. Specifically, SrmB is required to maintain a sufficient synthesis of r-proteins L13 and S9, failing which ribosomal subunit formation is impaired, resulting in consequent ribosomal and growth defects. Furthermore, the regulation of L13 and S9 synthesis occurs through a mechanism that involves the suppression of premature transcription termination by SrmB, a mode of regulation that has not been described for any DBP previously.

## MATERIALS AND METHODS

### Strains

The wt strain (MG1655*) used in this study is a derivative of MG1655 that contains a point mutation that restores the reading frame of the truncated *rph* gene in MG1655. Deletion alleles, marked with kanamycin resistance (kan^R^), were derived from the Keio strains collection (17) and transduced into MG1655* by PI transduction. As necessary, the kan^R^ marker was removed using FRT-recombinase (18). The *rne*1-702 and *rne*1-844 alleles with linked chloramphenicol-resistant (cam^R^) markers were derived from strains TM528 and TM527, respectively (19); the *rne*1-601 allele with a linked tetracycline-resistant marker was derived from strain BZ99 (20); the *rne1-498* allele was isolated in the lab as an insertion of the modified cam^R^ transposon λNK1324 immediately after *rne* codon 498 (21).

### Plasmids

Plasmid pTrc-L13 was made by amplifying the *rplM* coding region and the 5′ UTR using oligonucleotides with the sequence 5′ AAACCATGGACCCCACGTTACAAGAAAGTTTT 3′ and 5′ AAAAAGCTTCGATTAGATGTCAAGAACTTGCG 3′. The PCR product was digested with NcoI and HindIII and subcloned between the NcoI and HindIII sites of pTrc99c, downstream of the trc promoter. Plasmid pTrc-L13-S9 was made by amplifying the *rplM* and *rpsI* coding regions and the 5′ UTR using oligonucleotides with the sequence 5′ AAACCATGGACCCCACGTTACAAGAAAGTTTT 3′ and 5′ TTTCTGCAGTTACGCTGATTCAGATTTTAGC 3′. The PCR product was digested with NcoI and PstI and subcloned between the NcoI and PstI sites of pTrc99c. *rplM-lacZ* fusion plasmids were made by amplifying chromosomal DNA from wt or suppressor strains with oligonucleotides 5′ CGAAATGGCCTGCAACGTGCGC 3′ and 5′ TTTTGCTAGCCAGTTCAGTAGCCAGACG 3′. The resulting PCR products were digested with RsaI and NheI, yielding a product that contains 33 *rplM* codons and 439 bps of upstream DNA, which was subcloned between the SmaI and XbaI sites of pLACZY2 (22). A SrmB-expression plasmid was obtained from the ASKA collection (23), and a derivative containing a mutated DEAD-motif was made using a Quikchange mutagenesis kit (New England Biolabs).

### Identification and mapping of growth suppressors

Colonies of MG1655*Δ*srmB* were streaked on LB plates and incubated at 16°C for several rounds of growth until growth suppressors could be identified. Transposon mutagenesis was performed on the suppressor strains with λNK1324 to introduce random cam^R^ insertions into the chromosome. P1 phage was grown on pooled cam^R^ colonies and was used to transduce MG1655*Δ*srmB*, selecting for cam^R^ at 16°C. Colonies displaying a suppressor phenotype with a linked cam^R^ transposon were purified by restreaking. The location of chromosomal transposon insertion sites was determined by Sanger sequencing of chromosomal DNA derived from these strains.

### Single-copy *rplM-lacZ* translational fusions

The *rplM-lacZ* plasmids were recombined with λRS45 phage (24) and recombinant phage, identified by a blue color phenotype, were lysogenized into derivatives of the wt, Δ*srmB* or suppressor strains previously made *lacZ^−^* by transduction of a Δ*lacZ::kan* allele (17). β*-*Galactosidase activity was measured as described (25).

### Ribosome analysis

Ribosomes were analyzed by sucrose density ultracentrifugation of cell extracts using 14–32% sucrose gradients, as described (26).

### In vitro transcription reactions

Templates for transcription were made by PCR using oligonucleotides with the sequences 5′ GTTTGAGTTCCAGCGTTGCCTG 3′ and 5′ TAAAAGCTTACCCAATAAATAGTTAC 3′ and contained from 300 bp upstream of the *rplM* start codon to the end of the *rplM* 5′ UTR. Two-step transcription reactions were performed as follows: transcription was initiated in buffer containing 20 mM Tris, pH 8.0, 25 mM KCl, 2.5 mM MgCl_2_ and 0.5 mM DTT with 5 ng/μl of PCR product, 200 μM of ATP, CTP and GTP, and 0.25 units of *E. coli* RNA polymerase (New England Biolabs) Holoenzyme/μl. The reactions were incubated at 37°C for 15 min, stopped by the addition of one fourth volume of rifampicin (200 μg/ml), and placed on ice. Further elongation of a stalled product containing the first eight transcribed nts was performed by adding an equal volume of a cocktail containing 20 μM of CTP, GTP and UTP, 1 mM ATP, 0.1–0.2 μCi/μl of α-^32^P UTP and proteins, as indicated, in buffer containing 20 mM Tris, pH 8.0, 25 mM KCl and 0.5 mM DTT. The reactions were incubated at 16°C for 1–2 h, terminated by phenol extraction and the addition of loading dye, and resolved by electrophoresis on a 6% denaturing polyacrylamide gel. A ^32^P-labeled DNA ladder (New England Biolabs, cat # N3233S) was used as a size standard. As indicated, wild-type SrmB or a variant containing a mutated DEAD motif were added to transcription reactions after purification using the respective expression plasmids, as described (27).

### Mass spectrometry

Δ*lysA* derivatives of wt and Δ*srmB* strains were grown in Rich defined medium (Teknova) containing either ^12^C6, ^14^N2 lysine or a heavy atom derivative (^13^C6, ^15^N2). Cell extracts prepared from these strains were mixed in equal amounts and digested with trypsin. Liquid-chromatography-tandem mass spectrometry (LC-MS/MS) was performed using a Orbitrap Fusion Tribrid Mass Spectrometer at the Scripps Florida Proteomics Facility. The peak heights of peptides containing light and heavy-atom derivatives of lysine were used to quantify the relative levels of r-proteins in the wt and Δ*srmB* strains. To correct for any differences in sample preparation, the relative heavy to light atom r-protein ratios were normalized by the abundance of primary r-proteins (S4, S7, S8, S15 and S20 for the SSU; L4, L20, L22 and L24 for the LSU).

### RNA analysis

Total RNA was isolated using the hot-phenol method. For Northern-blot analysis, RNAs were fractionated on a 2% agarose gel in MOPS buffer containing 0.66 M formaldehyde or on a denaturing 6% polyacrylamide gel. After electrophoresis, the RNA was transferred to positively charged membrane (Nytran), cross-linked using 254 nM ultraviolet light, and hybridized with radiolabeled oligonucleotide probes for *rplM* mRNA (5′- TTCGAATAGCCTATGCCAGCACACAAAAA-3′) or *rplK* mRNA (5′- ATTATAAATTCCTCAAGTTGGG -3′). For the experiment shown in Figure 5D, a labeled riboprobe complementary to the first 130 nts of the *rplM* RNA was used instead. 3′-end RACE was performed by ligating total RNA derived from a Δ*pnp* strain with a pre-adenylated universal miRNA cloning linker (NEB cat #S1315S) using T4 RNA ligase 2 truncated KQ (NEB cat #S0373S). The reaction products were annealed with primer CJ1341 (5′ CCGTGATTGATGGTGCCTACAG 3′), which is complementary to the cloning linker, and reverse-transcribed. The cDNA products were amplified with CJ1341 and a forward DNA primer that corresponds to the start of the *rplM* mRNA (5′ GACCCCACGTTACAAGAAAGTTTTTTTC 3′). The resulting products were gel-purified and sequenced.

### SHAPE

8 μg of total RNA was cooled from 65°C to 35°C over 1 h with 5′ end-labeled CJ1082 (5′ GCCAGTTCAGTAGCCAGACGGC 3′) in 5 μl of Primer Extension buffer (10 mM Tris, pH 7.5, 75 mM KCl, 3 mM MgCl_2_). One tenth volume of DMSO or either 30 or 130 mM NMIA in DMSO was added to each tube and the tubes were incubated for 30 min at 37°C. Primer extension was performed by the addition of dNTPs and MMLV reverse transcriptase (28), and the reaction products were fractionated on a 6% denaturing polyacrylamide gel alongside radiolabeled sequencing products.

### Western-blot analysis

Fractions from sucrose gradients were collected and proteins were precipitated by TCA as described (12). Pellets were resuspended in loading buffer and proteins were separated on 4–20% TGX polyacrylamide gels (Biorad) and then transferred to nitrocellulose membranes. Membranes were incubated with polyclonal sheep antibodies against L13 and L2 r-proteins (a gift from R. Brimacombe, Max-Planck Institute, Berlin). Blots were decorated with horseradish peroxidase (HRP)-conjugated secondary antibodies (Sigma) and signals were revealed by ECL detection reagent (GE Healthcare). The chemiluminescent signal was revealed and quantified using the ImageQuant LAS4000 system (GE Healthcare). To measure L13 levels in whole-cell lysates, cell pellets were resuspended in loading buffer and analyzed as described above.

## RESULTS

### Isolation of Δ*srmB* low-temperature growth suppressors

Based on the *cs* phenotype of Δ*srmB* strains, we reasoned that suppressors of this phenotype, if identified, might yield new insights into the mechanism by which SrmB regulates ribosomal assembly. To identify such growth suppressors, Δ*srmB* strains were streaked on agar plates and incubated at 16°C. After several rounds of restreaking, individual colonies that exhibited enhanced growth at low temperatures were obtained. To map the mutations that confer growth suppression, random transposon mutagenesis was performed on each suppressor strain to insert a chloramphenicol-resistance (Cm^r^) marker at different locations on the chromosome. A subset of these insertions was found to exhibit a linkage between the Cm^r^ marker and the suppressor phenotype. Using transposon-specific sequencing primers, the chromosomal location of these linked insertions was determined. Two classes of transposon insertions were identified in this manner.

### Class I suppressors map to the *rne* gene

**Figure 1.**
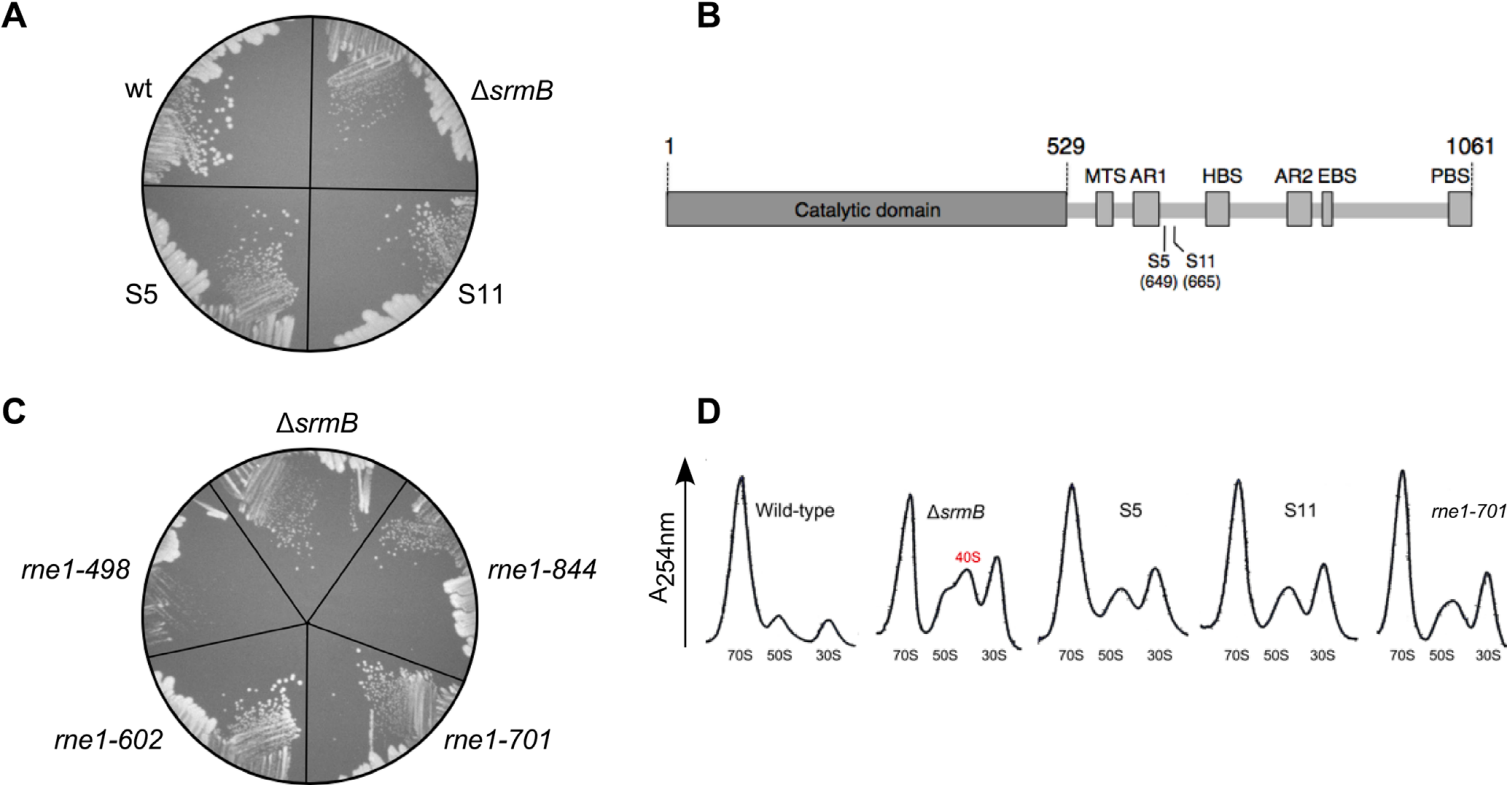
Class I suppressors of the Δ*srmB* cold sensitive defect map to the *rne* gene. (**A**) Two chromosomal suppressors that map to the *rne* gene (S5 and S11) were streaked on an LB-agar plate and incubated at 16°C for one week along with the wt and Δ*srmB* strains. (**B**) Schematic description of RNase E and the location of the Δ*srmB* suppressors. The non-catalytic region of RNase E (residues 530-1061) contains motifs responsible for the interactions with the inner membrane (MTS, residues 565–582), with RNA (arginine-rich RNA binding regions AR1 and AR2; residues 604–644 and 796–814, respectively) and with proteins that form the degradosome (HBS, RhlB helicase binding site, 719–731; EBS, enolase binding site, 834–850 and PBS, PNPase binding site, 1021–1061) (44). (**C**) Δ*srmB* strain derivatives that harbor different RNase E C-terminal deletions were streaked on an LB plate and incubated at 16°C along with a Δ*srmB* strain. The RNase E amino-acids present in each mutant are indicated. (**D**) Ribosomal profiles. The wt and Δ*srmB* strains, the S5 and S11 suppressors, and a Δ*srmB* strain derivative encoding RNase E amino-acids 1–701 were grown to midlog phase in LB medium at 16°C. Cell extracts derived from these strains were layered onto a sucrose gradient and ultracentrifuged. Ribosome profiles were generated at 254 nM. The 70S ribosomes and the 30S and 50S subunits are indicated. A 40S particle that accumulates in the Δ*srmB* strain is marked in red.

One group of insertions (Class I), obtained for two suppressor mutations (S5 and S11), exhibited a modest degree of growth suppression and was found to map to minutes 23–25 of the chromosome (Figure 1A). This region includes the *rne* gene encoding RNase E, a global regulator of RNA metabolism (29). Sequencing of the *rne* coding region revealed that each suppressor contains either a stop-codon (S5) or a frameshift mutation (S11) located within the C-terminal half of RNase E (Figure 1B). This region includes a scaffold that is required to form the RNA degradosome, an RNA-degrading protein complex that contains three additional proteins: the exoribonuclease, Polynucleotide phosphorylase (PNPase), the RNA helicase, RhlB and the glycolytic enzyme, Enolase. The RNase E variants in the S5 and S11 strains, therefore, are incapable of forming a functional degradosome.

To verify that a loss of the scaffold region is sufficient to confer a suppressor phenotype, *rne* mutations that remove different extents of the RNase E C-terminus were introduced into a Δ*srmB* strain. Similar to the Class I suppressors, deletion mutations that removed the entire scaffold region (*rne1-602* and *rne1-701*) grew better than the parental Δ*srmB* strain at 16°C, whereas a C-terminal deletion mutation (*rne1-844*) that incompletely removed the scaffold region did not (Figure 1C). Thus, the effect of the Class I suppressors appears to derive from an inability to form an intact RNA degradosome. A larger deletion variant (*rne1-498*) did not grow better than the parental Δ*srmB* strain, possibly because this more extensive deletion partly impairs the normal functions of RNase E inside the cell.

To test whether the suppressors also alleviate the ribosomal defects of Δ*srmB* strains at low temperatures, ribosomal profiles were generated from wt and Δ*srmB* strains, from the S5 and S11 suppressor strains, and from a reconstructed *rne* mutant (*rne 1-701*; Figure 1D). Ribosomal analysis revealed that the Δ*srmB* strain displayed elevated levels of ribosomal material within the subunit fractions compared to the wt strain. Specifically, significant amounts of incompletely matured LSU particles that sediment at 40S were found to accumulate in the Δ*srmB* strain, as has been observed previously (12). By comparison, each suppressor strain displayed a broad peak sedimenting at ∼45S comprised of reduced amounts of 40S particles and mature 50S particles. Nonetheless, the ribosomal profiles remained significantly perturbed in comparison to the wt strain, indicating that the *rne* suppressors do not fully restore efficient LSU assembly.

As a global regulator of RNA metabolism, RNase E has a pivotal role in multiple processes, including RNA turnover, stable RNA processing and small RNA regulation (29). We speculate that via one or more of its myriad functions, it regulates the expression of genes that affect the low-temperature growth of Δ*srmB* strains through its degradosome function. However, since the *rne* suppressor mutations still exhibited significant residual ribosomal defects, additional studies on the Class I suppressors were not pursued further.

### Class II suppressors map to the *rplM* gene encoding r-protein L13

**Figure 2.**
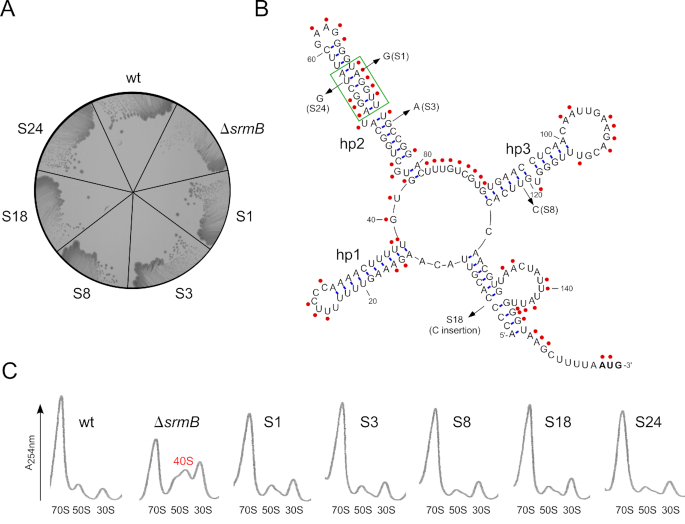
Class II suppressors of the Δ*srmB* cold sensitive defect map to the *rplM* gene. (**A**) Five Class II suppressors (S1, S3, S8, S18 and S24) were streaked on an LB-agar plate and incubated at 16°C along with a wt and a Δ*srmB* strain. (**B**) Location of the *rplM* suppressors and *rplM* 5′ UTR structure. The nucleotide changes corresponding to each suppressor is shown on the computationally folded structure of the *rplM* 5′UTR using Mfold (45). The three hairpin-loop structures proposed to form within the UTR (hp1–hp3) are indicated, and the initiation codon is shown in bold lettering. Single stranded regions within the RNA, shown by red circles, were experimentally determined by treating total cellular RNA with NMIA followed by primer extension using a *rplM*-specific oligonucleotide. A region displaying notable differences in RNA conformation between the experimental data and the computational predictions is enclosed within a green rectangle. (**C**) Ribosome profiles. Ribosome profiles for the wt and Δ*srmB* strains, as well for each of the Δ*srmB* Class II suppressors, were determined as described in the Figure 1D legend. A 40S peak that is present in Δ*srmB* strains, but significantly reduced in each of the suppressor strains, is also marked.

A second class of suppressors (Class II) exhibited an enhanced degree of growth suppression (Figure 2A) and was found to map to minutes 72–73 of the chromosome. This region includes the gene for r-protein L13 (*rplM*), which was previously shown to be absent in 40S particles isolated from Δ*srmB* strains (12). Notably, L13 is one of five r-proteins required to initiate LSU assembly (30) and the only such protein that is absent in the 40S particle. Sequencing of the *rplM* region of the chromosome revealed that each suppressor maps to the *rplM* 5′ UTR, suggesting a regulatory role in L13 expression (Figure 2B).

An *in silico* analysis of the *rplM* 5′ UTR using RNA folding software (Mfold) suggested that the RNA is highly structured, forming three internal hairpin-loop structures (hp1–hp3), as well as a long-distance stem formed by pairing of the 5′ and 3′ regions of the UTR (Figure 2B). The secondary structure of the RNA was experimentally determined by using a single-strand specific RNA modification reagent N-methyl isatoic anhydride (NMIA), and was found to broadly agree with the computationally predicted structure and a recently determined *in vivo* structure (31). Three suppressor mutations (S1, S3 and S24) were found to map to hp2, whereas one mutation (S8) mapped to hp3 and another (S18) to the stem formed by the two ends of the 5′ UTR.

To determine whether Class II suppressors rescue ribosomal defects, ribosomal profiles were generated for each suppressor strain at 16°C (Figure 2C). In each case, the ribosomal profile was found to closely match the profile of the wt strain. In particular, the accumulation of 40S particles was nearly abolished. Thus, the Class II suppressors significantly reverse the ribosomal defects exhibited by Δ*srmB* strains.

### The *rplM* suppressors regulate L13 expression

**Figure 3.**
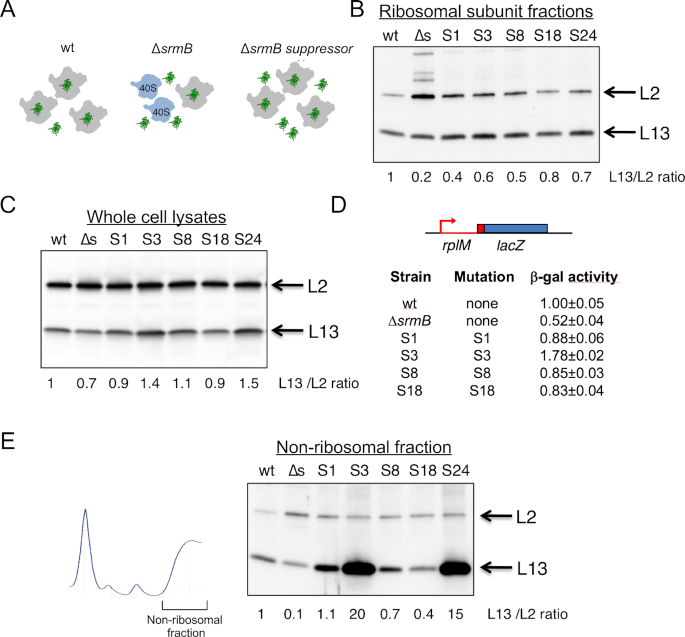
Expression of *rplM* is reduced in Δ*srmB* strains. (**A**) Model for suppression of the Δ*srmB* cold-sensitive defect by L13 over-expression. Left, LSU assembly in a wt strain. L13, shown in green, is incorporated into the mature LSU (shown in grey); middle panel, an absence of SrmB is proposed to prevent the incorporation of L13 into LSUs, resulting in the formation of immature 40S particles (shown in blue); right, L13 over-expression results in the forced incorporation of L13 into LSUs when SrmB is absent. (**B**) Western-blot analysis of L13 levels in ribosomal subunits. Ribosomal subunit fractions (from 30S to 50S) corresponding to the profiles shown in Figure 2C were collected and r-proteins L13 and L2 were analyzed by Western blot. The numbers at the bottom represent the relative concentration of L13 in ribosomal particles, after normalization to L2, for the representative blot. The mean values of the relative L13/L2 ratio obtained from two to three experiments are 1 (wt), 0.3 (Δ*srmB*), 0.6 (S1), 1.2 (S3), 0.6 (S8), 0.8 (S18) and 0.8 (S24). (**C**) Western blot analysis of L13 levels in whole-cell lysates. Total cell extracts prepared from the strains indicated in (B) were analyzed by western blot using antibodies to L13 and L2. The L13/L2 ratios, relative to the wt strain, are denoted for the blot shown. The mean values obtained from four experiments are 1 (wt), 0.7 (Δ*srmB*), 0.9 (S1), 1.3 (S3), 1 (S8), 0.9 (S18) and 1.4 (S24). (**D**) Activity of *rplM-lacZ* fusions. A *rplM-lacZ* translation fusion containing a *rplM* cassette that includes its promoter, an unmutated 5′ UTR or one containing the indicated mutations, and the first 33 codons (in red) was fused to the *lacZ* coding region (in blue), and integrated into the chromosome of Δ*lacZ* derivatives of wt, Δ*srmB* or the indicated suppressor strains. The *β*-galactosidase activity of the derivative strains was determined after growth at 16°C and normalized with respect to the wt strain. The mean values and standard errors are based on four measurements for each strain. (**E**) Western-blot analysis of L13 levels in non-ribosomal fractions. The bulk fraction at the top of the gradients was collected and ribosome-free r-proteins L13 and L2 were analyzed by Western blot. Left: Ribosome profile depicting the fraction collected for analysis; right: the fractions were analyzed by Western blot using antibodies to L13 and L2. The numbers below represent the relative L13/L2 ratio for the blot shown. The mean values obtained from two to four experiments are 1 (wt), 0.13 (Δ*srmB*), 1.1 (S1), 31 (S3), 0.6 (S8), 0.4 (S18) and 10 (S24).

Given the previously noted deficit of L13 in 40S particles and the canonical viewpoint that DBPs function in ribosome assembly by reorganizing rRNA, our initial hypothesis to explain the previously noted L13 deficit in 40S particles was that SrmB’s function in ribosome assembly is to confer structural changes on the LSU to facilitate L13 binding (Figure 3A). Lacking SrmB, ribosomal incorporation of L13 is hampered resulting in the observed accumulation of 40S particles. A plausible mechanism for suppressor function, by this model, would be that the suppressors cause high-level over-expression of L13, resulting in the forcible incorporation of L13 into the LSU despite a sub-optimally configured L13 binding site.

To test the different predictions of this model, we first investigated whether L13 incorporation into the LSU was restored in the suppressor strains. Ribosomal proteins from subunit fractions were separated by SDS-PAGE and L13 was quantified by Western blot analysis. As a control, L2 levels were also analyzed, since this r-protein was found present at normal amounts in *ΔsrmB* 40S/50S particles (12). We observed that L13 levels in Δ*srmB* subunit fractions were reduced by 70–80% (Figure 3B), consistent with previous observations (12). Significantly, fractions derived from the Class II suppressor strains were each found to display increased L13 levels, consistent with the model in Figure 3A.

Next, to determine whether L13 is over-expressed in the suppressor strains, L13 levels were measured in whole-cell lysates. Surprisingly, a high degree of L13 over-expression was not observed (Figure 3C). A ∼1.5-fold increase was observed for S3 and S24 mutants, whereas for the other suppressors L13 levels were comparable to the wt strain. Because the ribosomal defects of the *ΔsrmB* strain could be suppressed without a significant increase in L13 expression, these data were inconsistent with the model presented in Figure 3A.

Interestingly, we noted that L13 levels in the *ΔsrmB* strain were modestly but significantly reduced compared to the wt strain, suggesting that L13 synthesis might be reduced in the former strain. To validate these results, chromosomal *rplM-lacZ* fusions, which are expected to reflect the L13 synthesis rate, were constructed. Significantly, the β-galactosidase activity of the *rplM-lacZ* fusion in the *ΔsrmB* strain was reduced to nearly half of the expression level in the wt strain (Figure 3D). A similar decrease (∼40%) of β-galactosidase activity was also observed when a wt *rplM-lacZ* fusion was transferred into each of the suppressor strains (data not shown), indicating that the reduced activity in the Δ*srmB* strain is not due to indirect effects such as slow growth or ribosome defects. As with the results obtained using Western-blot analysis, the introduction of the suppressor mutations into the *rplM-lacZ* fusions increased β-galactosidase activity in each case.

Although these data suggested that an absence of SrmB reduces L13 expression directly, one other potential explanation for this effect was based on the prior observation that *rplM* mRNA expression can be repressed when L13 is over-expressed (32). That observation raised the possibility that the initial model could alternatively explain the reduced levels of L13 observed because a failure to incorporate L13 into the LSU would result in an increased sub-population of free L13 (Figure 3A, middle panel), which then represses L13 synthesis via feedback regulation. If that were so, increased levels of free L13 would be expected to be observed in the Δ*srmB* strain. To test this possibility, L13 levels in the ribosome-free fraction were measured for the wt and Δ*srmB* strains (Figure 3E), However, free L13 was found to be present at a 10-fold lower level in the Δ*srmB* strain, rather than at elevated levels, indicating that the overall lower levels of L13 in the Δ*srmB* strain cannot alternatively be explained by feedback regulation. Of interest, the S3 and S24 suppressors, which modestly increase total L13 levels relative to a wt strain (Figure 3C), exhibited large increases in free L13, indicating that free L13 normally comprises a small fraction of total L13 in the cell whose abundance is amplified by changes in total L13 levels. Altogether, these results show that the absence of SrmB leads to reduced L13 synthesis and the suppressor mutations compensate for this defect by increasing L13 expression. A corollary of these results is that a molecular role of SrmB in ribosome assembly is to ensure that adequate levels of L13 are synthesized so that it does not become a bottleneck for ribosome synthesis.

### The r-protein S9 is also underproduced in Δ*srmB* strains

**Figure 4.**
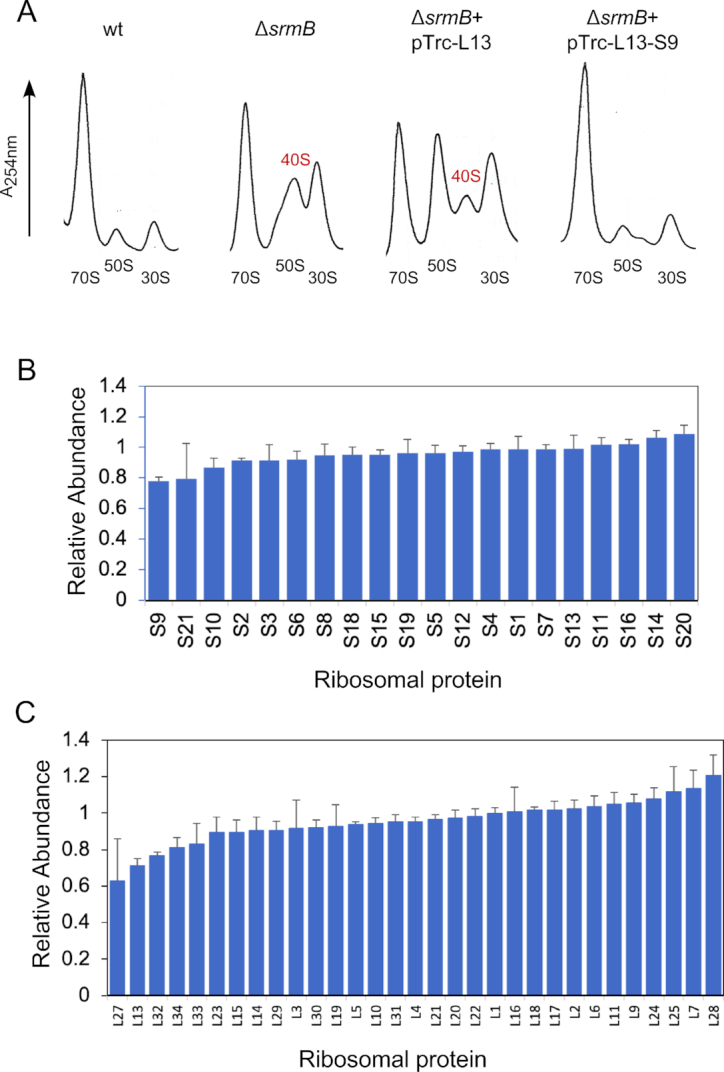
The level of the r-protein S9 is reduced in Δ*srmB* strains. (**A**) Ribosomal profiles. Ribosomal profiles were generated from a wt strain, a Δ*srmB* strain, or a Δ*srmB* strain containing plasmids expressing either L13 or both L13 and S9 after growth at 16°C. The 40S LSU intermediate is indicated. (**B**) Mass spectrometric analysis of SSU proteins in whole-cell lysates. *LysA* derivatives of wt and Δ*srmB* strains were grown in defined media containing light and heavy-atom derivatives of lysine at 16°C, respectively. Cell extracts derived from these strains were combined in equal proportions followed by quantitative Mass Spectrometry using SILAC (46,47). The peaks heights of peptides containing heavy and light atoms were used to quantify the relative levels of the different SSU proteins in the two strains. (**C**) Mass spectrometric quantitation of LSU protein levels in wt and Δ*srmB* strains. The relative levels of LSU proteins were quantified as in (B). The mean values and standard errors for each r-protein in (B) and (C) are derived from four SILAC experiments.

To confirm that a L13 deficit is responsible for the observed ribosomal defects of Δ*srmB* strains, we attempted to rescue these defects by transforming a Δ*srmB* strain with a multicopy L13-expressing plasmid. However, the ribosomal profile of the derivative strain displayed a significant residual accumulation of 40S particles (Figure 4A). We also noticed that the Δ*srmB* strain, with or without the L13 plasmid, has a high level of material in the 30S region, which corresponds to the ribosomal small subunit (SSU). This large peak could contain both mature and immature 30S particles, raising the possibility that SSU maturation could be defective in these strains as well.

Significantly, the *rplM* gene belongs to a bicistronic operon in which the second gene, *rpsI*, encodes the SSU r-protein, S9. We hypothesized that the Δ*srmB* ribosomal defects may be caused by a deficiency of both *rplM* and *rpsI*. Consistent with this hypothesis, when S9 was ectopically co-expressed with L13, a ribosomal profile that closely matched the wt profile, was observed (Figure 4A). These findings suggested that S9 expression is also reduced in the Δ*srmB* strain and that SrmB thereby regulates both genes in the *rplM-rpsI* operon.

To confirm that the synthesis of S9 is reduced in the Δ*srmB* strain, the relative abundance of SSU proteins was determined in whole cell lysates by using quantitative Mass Spectrometry (MS). Strikingly, S9 was found to be the most depleted SSU protein in the Δ*srmB* strain, being present at a 22% lower level than in the wt strain, confirming its reduced expression in the former strain (Figure 4B).

Quantitative MS analysis was also used to determine the relative abundance of the LSU proteins. Significantly, among the five LSU proteins required to initiate assembly, L13 was found to be reduced in the Δ*srmB* strain by nearly 30%, whereas each of the other such proteins (L4, L20, L22 and L24) was present in normal amounts (Figure 4C). These observations, along with those shown in Figure 3, confirmed the reduced synthesis of L13 in Δ*srmB* cells. Four other LSU proteins were also found to be depleted in the Δ*srmB* strain by >20% (L27, L32, L33 and L34). These proteins were also earlier found to be under-represented in the 40S particles purified from a Δ*srmB* strain (12) and may require prior L13 incorporation to be assembled into the LSU. Altogether, these results show that both L13 and S9 are under-produced in Δ*srmB* strains, leading to the accumulation of LSU and SSU intermediates that lack L13 and S9, respectively.

### SrmB reduces the formation of prematurely terminated transcription products

**Figure 5.**
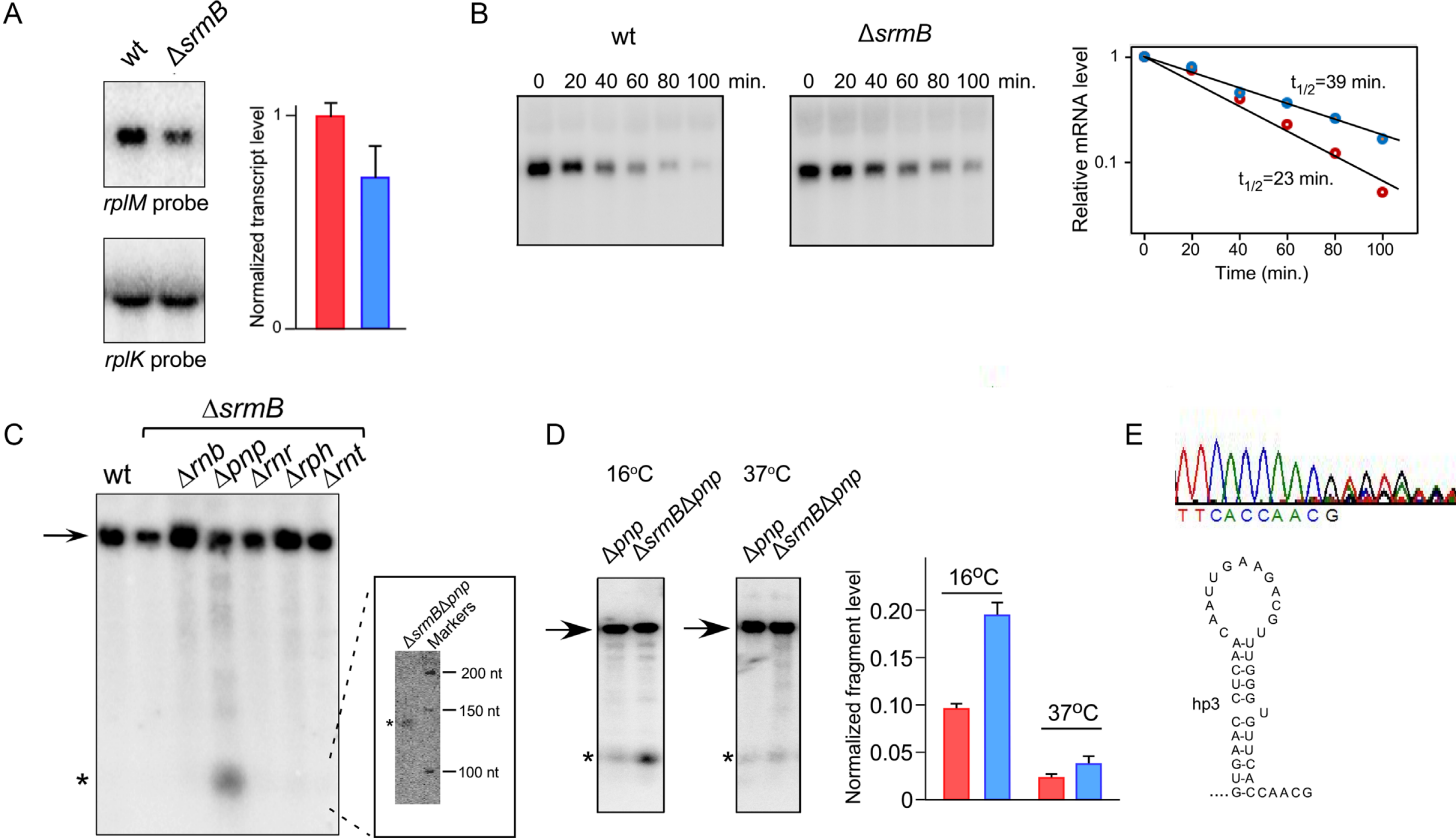
A prematurely terminated *rplM-*derived transcript accumulates in the absence of SrmB. (**A**) Northern-blot analysis of *rplM-rpsI* mRNA. Total RNA was isolated from wt and Δ*srmB* strains after growth at 16°C and analyzed by Northern blotting using an agarose gel and a labeled oligonucleotide probe complementary to the first 40 nts of the *rplM* 5′ UTR. This probe reveals a ∼1 kB transcript that corresponds to the full-length bicistronic mRNA. The levels of the *rplM-rpsI* mRNA were normalized to *rplKAJL* mRNA levels by re-probing the blot with a *rplK* probe. Red bars, wt strain; blue bars, Δ*srmB* strain. The mean values and standard errors are derived from seven measurements for each strain. (**B**) Half-life of the *rplM-rpsI* mRNA. Total RNA was isolated from the wt and Δ*srmB* strains grown at 16°C after the addition of rifampicin for various periods of time, as indicated. The RNAs were analyzed by Northern blotting, as described in (A). The levels of the *rplM-rpsI* mRNA were plotted on a semi-logarithmic graph and best fit straight lines were drawn through data points. Red, data for the wt strain; blue, data for the Δ*srmB* strain using mean values from three experiments. (**C**) Identification of a prematurely terminated *rplM* transcript. Northern blot analysis was performed on total RNA derived from wt or Δ*srmB* strains grown at 16°C, or from Δ*srmB* strain derivatives lacking different exoribonucleases. An arrow depicts the full length *rplM-rpsI* transcript, whereas, a prematurely terminated transcript fragment is denoted by an asterix. (Inset) northern blot analysis of RNA derived from the Δ*srmB*Δ*pnp* strain that was fractionated alongside radiolabeled DNA markers on a denaturing polyacrylamide gel. (**D**) Analysis of prematurely terminated *rplM-rpsI* transcripts in Δ*pnp* and Δ*srmB*Δ*pnp* strains. Total RNA was isolated from Δ*pnp* and Δ*srmB*Δ*pnp* strains after growth at either 16°C or 37°C, and analyzed by Northern blotting using a 5′ UTR *rplM* riboprobe. The levels of the prematurely terminated products were normalized to the levels of the full-length mRNA. Red bars, *srmB*^+^ strain background; blue bars, Δ*srmB* strain background. The mean values and standard errors are derived from 3–5 measurements for each strain. (**E**) 3′-end determination of the prematurely terminated *rplM* transcript. The prematurely terminated *rplM* transcript was gel eluted and analyzed by 3′ RACE. The sequence trace corresponding to the 3′ end of the fragment is shown at the top. The bottom depicts hp3 and the downstream sequences that can be resolved in the sequence trace.

The reduced expression of L13 and S9 in absence of SrmB (Figures 3 and 4) suggested that SrmB positively regulates both *rplM* and *rpsI* expression. Consistent with an RNA remodeling function for this DBP, SrmB could be regulating the level and/or the translation of the *rplM-rpsI* transcript. To distinguish between these possibilities, *rplM-rpsI* mRNA was analyzed by Northern blot using total RNA extracted from the wt and Δ*srmB* strains. As a control, the amount of *rplKAJL* mRNA was also analyzed. A quantitation of the *rplM-rpsI* mRNA indicated a reduction of ∼30% in the Δ*srmB* strain (Figure 5A). Notably, this decrease was similar in magnitude to that observed for L13 and S9 by both Western Blot and MS analysis (Figures 3 and 4), suggesting that the effect of SrmB on L13 and S9 synthesis is mediated through changes in the levels of the full-length *rplM-rpsI* mRNA.

To determine whether the decreased amount of the *rplM-rpsI* mRNA in the Δ*srmB* strain could be due to reduced mRNA stability, the rate of *rplM-rpsI* decay was measured in wt and Δ*srmB* strains grown at 16°C. Cultures were treated with rifampicin to block transcription and RNA was isolated at different times and analyzed by Northern Blot (Figure 5B). Contrary to expectation, the *rplM-rpsI* transcript was found to exhibit a 60% stability increase in the Δ*srmB* strain, rather than decreased stability. Thus, the lower amounts of *rplM-rpsI* mRNA in Δ*srmB* cells cannot be explained by enhanced degradation.

Next, we tested whether the SrmB-dependent changes in mRNA levels can instead be explained by premature transcription termination. However, since preliminary Northern blot analyses did not show any evidence for a prematurely terminated product, we reasoned that such a product might be rapidly degraded. Thus, Northern blot analysis was repeated on derivatives of the Δ*srmB* strain lacking each of the prominent *E. coli* exo-RNases. No truncated product was observed in strains lacking RNase II, RNase R, RNase PH or RNase T, but in a strain lacking PNPase, a low level of a small RNA product could be seen (Figure 5C). This result suggested that some premature transcription termination occurs during the synthesis of the *rplM-rpsI* mRNA generating a small product that is degraded in a PNPase-dependent manner. To determine the size of this product more accurately, RNA from a Δ*srmB*Δ*pnp* strain was analyzed by Northern blot using a higher resolution polyacrylamide gel (Figure 5C inset), which indicated a size of ∼140 nts.

We then hypothesized that if SrmB regulates *rplM-rpsI* mRNA expression by suppressing premature transcriptional termination, the amount of the terminated product should be lower in a wt strain relative to a Δ*srmB* strain at 16°C. Moreover, given that reduced ribosomal defects are observed in Δ*srmB* strains at 37°C (12,26), the extent of the termination product formation should be decreased at this higher temperature. To test these predictions, RNA was isolated from Δ*pnp* derivatives of wt and Δ*srmB* strains after growth at either 16°C or 37°C and analyzed by Northern blotting (Figure 5D). Significantly, higher levels of the termination product were found to accumulate in the Δ*srmB* strain at 16°C. Thus, while the relative levels of the *rplM-rpsI* full-length mRNA decreased in the Δ*srmB* strain (Figure 5A), the small RNA product was increased, suggesting a precursor-product relationship. Additionally, the levels of the small RNA product were also lower upon growth at 37°C, both in the wt and the Δ*srmB* strains. Furthermore, the differences in the levels of these products were significantly reduced at the higher temperature, as compared to 16°C. Overall, the identification of a prematurely terminated product, whose abundance changes in a predictable manner, is consistent with a positive effect of SrmB in reducing premature transcript termination within the *rplM-rpsI* operon.

Finally, to determine the 3′ terminus of the prematurely terminated product, an adaptor was ligated to the 3′ end of the gel-eluted RNA product and its sequence was determined by Rapid Amplification of cDNA Ends (RACE). Unambiguous sequences up to five nts beyond hp3 could be clearly discerned at the end of the sequence trace (Figure 5E), but downstream of this sequence a mixed base composition was observed. This pattern suggests that termination occurs at least five nts downstream of hp3 with heterogeneity being caused either by termination occurring at multiple positions or by residual 3′-end processing.

### Recapitulating premature *rplM-rpsI* transcription termination *in vitro*

**Figure 6.**
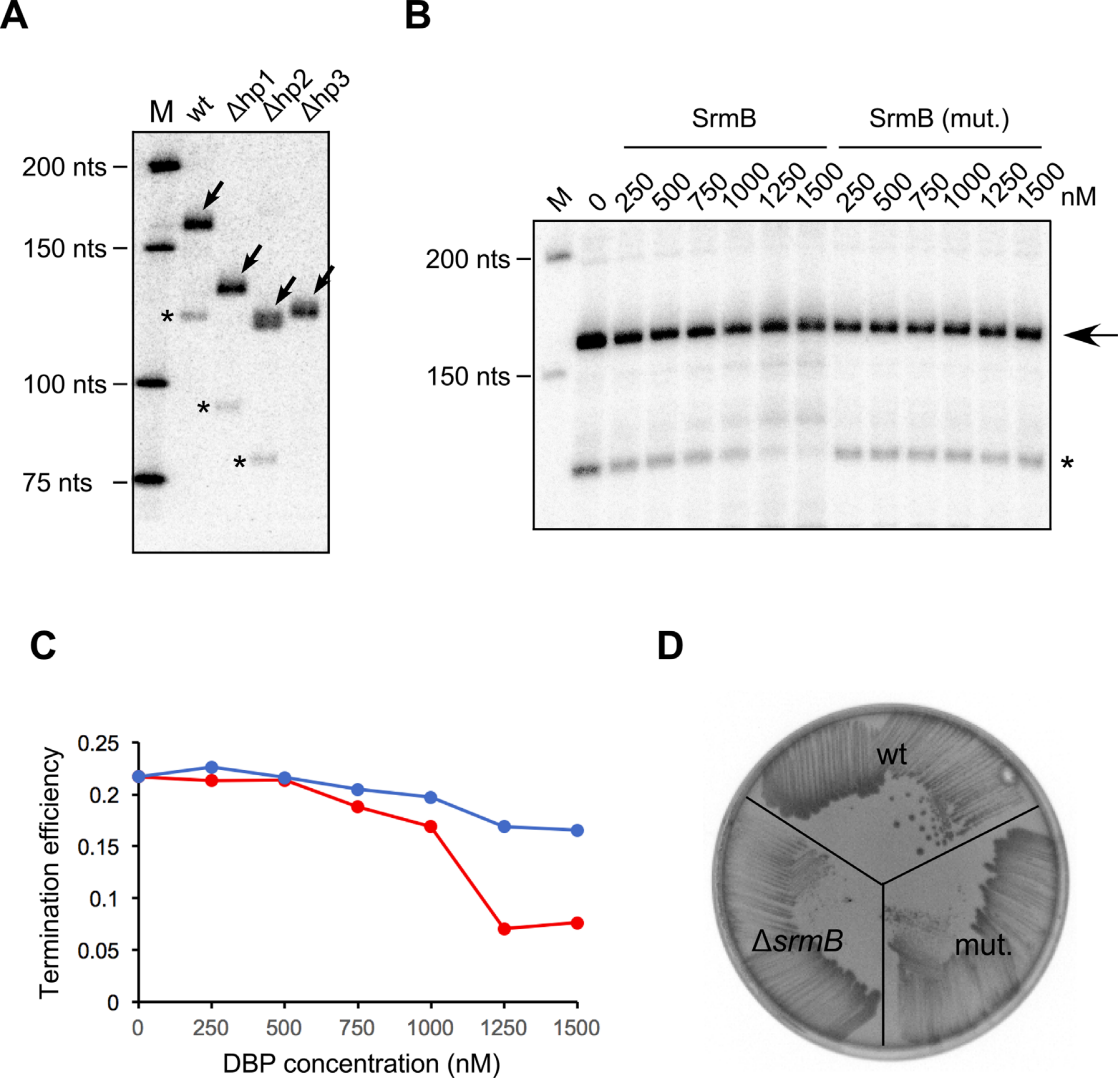
Recapitulating SrmB-dependent premature transcription termination within the *rplM* 5′UTR *in vitro*. (**A**) PCR products containing the *rplM* promoter and either the entire *rplM* 5′ UTR, or mutant versions lacking hp1, hp2 or hp3, were added to transcription reactions containing *E. coli* RNA polymerase Holoenzyme, buffer and NTPs, including radiolabeled UTP. The transcription reactions were resolved by denaturing polyacrylamide gel electrophoresis. Arrows denote runoff transcription products; prematurely terminated products are denoted by an asterix. M, ^32^P labeled low molecular weight DNA marker. (**B**) Effect of SrmB on premature transcription termination. Transcription reactions using the wt DNA template, as described in (A), were performed in the absence or the presence of the indicated concentrations of wt SrmB or an ATPase defective variant. The runoff and termination products are depicted by an arrow and an asterix, respectively. (**C**) Quantitation of termination products. The fraction of the termination products observed in (B) was normalized to the levels of the runoff transcript and plotted as a function of DBP concentration present in the reaction. Red, termination efficiency with different concentrations of wt SrmB; blue, termination efficiency with the ATPase-defective SrmB variant. Mean values are derived from four experiments. (**D**) Mutation of the SrmB DEAD motif confers cold-sensitivity. A strain derivative containing the SrmB DEAD>AAAD mutation (mut.) was streaked on an LB-agar plate along with a wt and a Δ*srmB* strain and incubated at 16°C.

We were next interested in determining whether the premature transcription termination products observed *in vivo* can be generated *in vitro*. To do so, a transcription reaction was established using purified *E. coli* RNA polymerase holoenzyme and a PCR product that included the *rplM* promoter and the 5′ UTR. An initial set of reactions using standard transcription conditions yielded the expected runoff transcript but no prematurely terminated products (data not shown). It is known that the efficiency of many terminators *in vitro* is sensitive to buffer conditions, and in particular, effective termination often requires low Mg^2+^ concentrations (33). However, transcription initiation is inefficient at low Mg^2+^ concentrations. Therefore, to enable transcription termination under low Mg^2+^ concentrations, a two-step procedure was developed. In this procedure, transcription was initiated with three NTPs (ATP, CTP and GTP) in the presence of 2.5 mM Mg^2+^ concentration, followed by the dilution of the stalled elongation complexes into a mixture containing all four NTPs and 1 mM Mg^2+^ final concentration. Under these conditions, a moderate amount of prematurely terminated product could be seen (Figure 6A), indicating that the transcription termination that is observed *in vivo* can be recapitulated *in vitro* without the addition of any other factors. Moreover, to determine whether any structural features in the *rplM* 5′ UTR are necessary for termination, DNA templates lacking hp1, hp2 or hp3 were also tested. Termination products were observed with templates lacking hp1 or hp2, but not without hp3. Thus, hp3, which is just upstream of the 3′ end of the prematurely terminated product (Figure 5E), appears to be important for premature termination. It has been recently shown that L13 binds to the initial part of the *rplM* transcript *in vitro*, but binding is abolished upon hp1 deletion, suggesting that hp1 could function as a binding element for this protein (31). Whether L13 binding to hp1 affects SrmB function on the *rplM* mRNA is not known.

We then determined whether the transcription termination observed *in vitro* can be suppressed by addition of SrmB. For this purpose, different concentrations of purified SrmB was added to the transcription reactions during the elongation step. As a control, an SrmB variant that contains two mutations in the SrmB DEAD motif (DEAD>AAAD), which reduces the *in vitro* ATPase activity of SrmB to ∼1% of the wt protein, was also used. Decreased levels of the terminated transcript were observed with increasing SrmB concentration, consistent with a direct effect of SrmB on premature termination (Figure 6B and C). In contrast, the SrmB variant showed a lower degree of decrease, suggesting that the ATPase activity of SrmB is important to suppress premature transcription termination. To determine whether the ATPase activity of SrmB is also required for its effects on cell growth, the DEAD > AAAD mutation was incorporated into the genomic *srmB* locus. When grown at 16°C, this strain exhibited a cold-sensitive growth phenotype similar to the SrmB-deletion mutant, suggesting that the ATPase activity of SrmB is also required for ribosomal function *in vivo* (Figure 6D).

### The suppressor mutations reduce the formation of prematurely terminated transcription products

**Figure 7.**
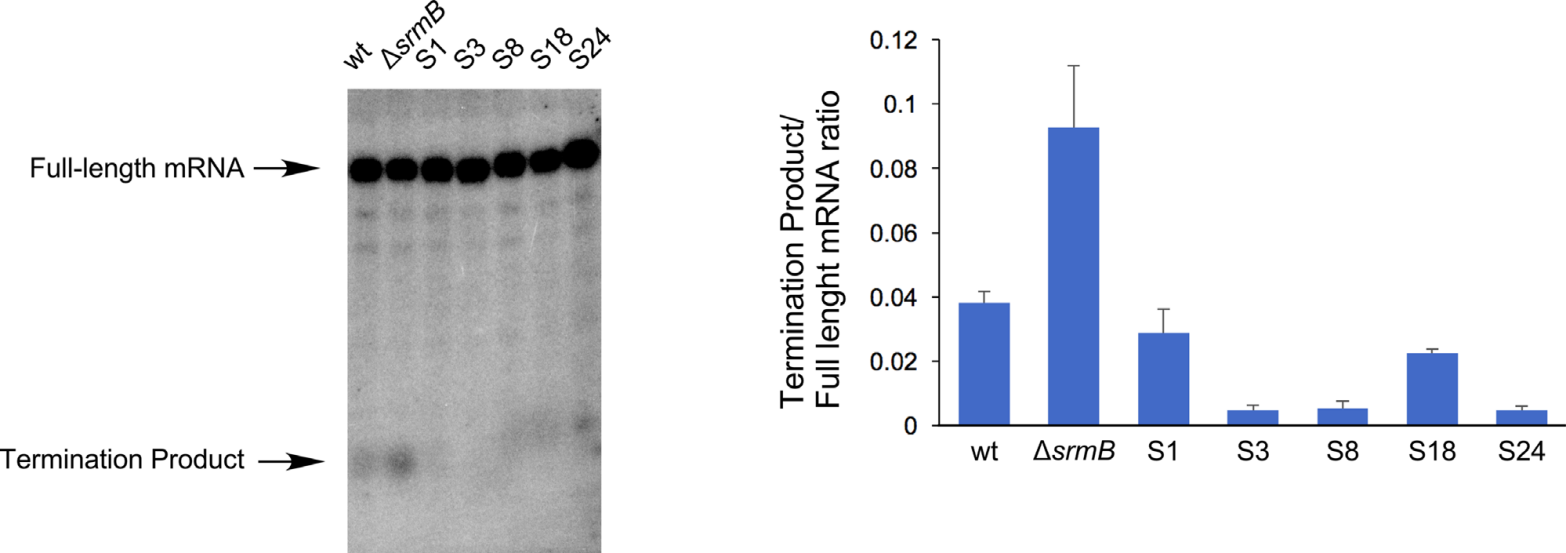
The Δ*srmB* suppressors reduce premature transcription termination. The levels of full-length *rplM-rpsI* mRNA and the prematurely terminated transcription product were evaluated by Northern blot analysis using RNA derived from Δ*pnp* derivatives of wt, Δ*srmB* or suppressor strains grown at 16°C. The levels of the termination product, relative to the full-length mRNA, is depicted. Mean values and standard errors are derived from three measurements for each strain.

Finally, to analyze the basis for the reduced cold-sensitivity of the Class II suppressor mutants (Figure 2A), we compared the amounts of prematurely terminated transcript in Δ*pnp* derivatives of the wt, Δ*srmB* and the suppressor strains. Significantly, a decrease in the levels of this product was observed for each suppressor strain (Figure 7). Of note, the S3 and S24 strains, which yielded the highest levels of *rplM* expression (Figure 3C–E), showed the lowest degree of premature transcription termination. Thus, the basis for the function of the suppressor mutants appears to stem from a reversal of the enhanced premature transcription termination that is observed in the Δ*srmB* strain.

Given that termination appears to function through a hp3-dependent mechanism (Figure 6A), it is surprising that four of the five suppressor mutations lie outside of hp3 (S1, S3, S18 and S24). We propose that the effects of the suppressor mutations are transmitted through interactions with hp3 or other, yet to be identified, determinants important for transcription termination. Notably, S1, S3 and S24 lie within or close to a region of the *rplM* 5′ UTR that was found by SHAPE to display structural fluidity, which might therefore interact with sequences important for termination (Figure 2B). Further work on this topic will be required to delineate the precise basis by which these distal mutations influence transcription termination, and thereby, suppress the Δ*srmB cs* phenotype.

## DISCUSSION

DBPs represent a well-established class of RNA remodeling factors with many members known to play important roles in ribosome assembly (13,34–36). It has been proposed that DBPs function in this process by remodeling rRNAs or rRNA–protein interactions to facilitate snoRNA–rRNA interactions, enable pre-rRNA processing or promote the acquisition of a native rRNA conformation. Nonetheless, the precise molecular basis for the ribosomal function of these proteins is not well understood.

To further define the role of the *E. coli* SrmB DEAD-box protein in ribosome assembly, we adopted a genetic approach, whereby growth suppressors of the cold-sensitive growth phenotype of Δ*srmB* strains were identified. Two classes of suppressors, mapping to either the *rne* or the *rplM* locus, were found. The *rne* gene encodes RNase E, a major *E. coli* RNase, and we hypothesize that the suppressing mutations in RNase E confer cold-resistance through a mechanism that involves altered degradation of RNase E substrates. The second set of suppressors, which displayed a more pronounced suppressor phenotype, was found to map to the *rplM* 5′ UTR. This gene encodes the r-protein L13, which was previously shown to be absent in the 40S particles that accumulate in absence of SrmB (12). Each of these suppressors corrected both the Δ*srmB* growth and ribosome assembly defects, and the incorporation of L13 into the subunits was increased (Figure 2A, C and 3B). Moreover, we found that the synthesis of L13 was reduced in the Δ*srmB* strain and restored in the suppressor strains (Figure 3C and D). Furthermore, we also showed that the S9 r-protein, which is expressed from the *rplM-rpsI* operon, was found at reduced levels in the Δ*srmB* strain (Figure 4B). Thus, our results show that the growth and ribosome assembly defects of Δ*srmB* are linked to a deficit of both r-proteins L13 and S9, which was confirmed by the rescue of ribosome assembly defects following ectopic overexpression of L13 and S9 (Figure 4A). We propose that in the absence of SrmB, the extent of L13 and S9 synthesis is insufficient to meet the requirements of growing cells, resulting in the accumulation of LSU and SSU particles that lack these proteins, as well as those whose assembly depends upon the prior incorporation of these two proteins.

To identify the mechanism responsible for reduced L13 and S9 synthesis in the Δ*srmB* strain, we analyzed the *rplM-rpsI* mRNA by Northern blot. In cells lacking SrmB, full-length *rplM-rpsI* transcript levels were found to be reduced by ∼30% at low temperatures (Figure 5A), closely paralleling the reduction in the levels of L13 and S9 synthesis determined by quantitative Western Blot and MS analyses (Figures 3 and 4). Notably, the growth rate of the Δ*srmB* strain at 16°C was previously found to be reduced by 30–35% compared to the wt strain (37), suggesting that the reduced synthesis of L13 and S9 is limiting for cell growth. Conversely, the appearance of a prematurely terminated product, whose amount was significantly increased in the Δ*srmB* strain, was observed (Figure 5D). The inverse correlation between the levels of the full-length and the prematurely terminated product suggested a mechanism whereby full-length *rplM-rpsI* transcript synthesis is compromised by premature transcription termination. Collectively, these data converge on the notion that the synthesis level of L13/S9 is rate limiting for both ribosome assembly and cell growth at low temperatures and that SrmB acts as a positive regulator of L13/S9 synthesis by abrogating premature transcription termination. Strikingly, these findings also illustrate how modest changes in r-protein synthesis (Figure3C and D) can significantly perturb ribosome assembly (Figures 2C and 4A).

To confirm the proposed molecular function of SrmB on *rplM-rpsI* mRNA synthesis, transcription reactions were performed *in vitro* and a termination product similar to that observed *in vivo* was observed (Figure 6). The formation of such product without the addition of other factors suggests a Rho-independent mode of transcription termination (38), which only requires *E. coli* RNA polymerase and transcription termination determinants encoded within the DNA template. Consistent with such a mode of termination, which requires a hairpin structure to elicit RNA polymerase dissociation, no such termination products were observed when hp3, the hairpin closest to the site of transcription termination, was deleted. Additional transcription analyses showed that the addition of SrmB to the transcription reactions resulted in a dose-dependent reduction of the amount of the prematurely terminated product, indicating that the presence of this DBP is sufficient to mediate this process.

**Figure 8.**
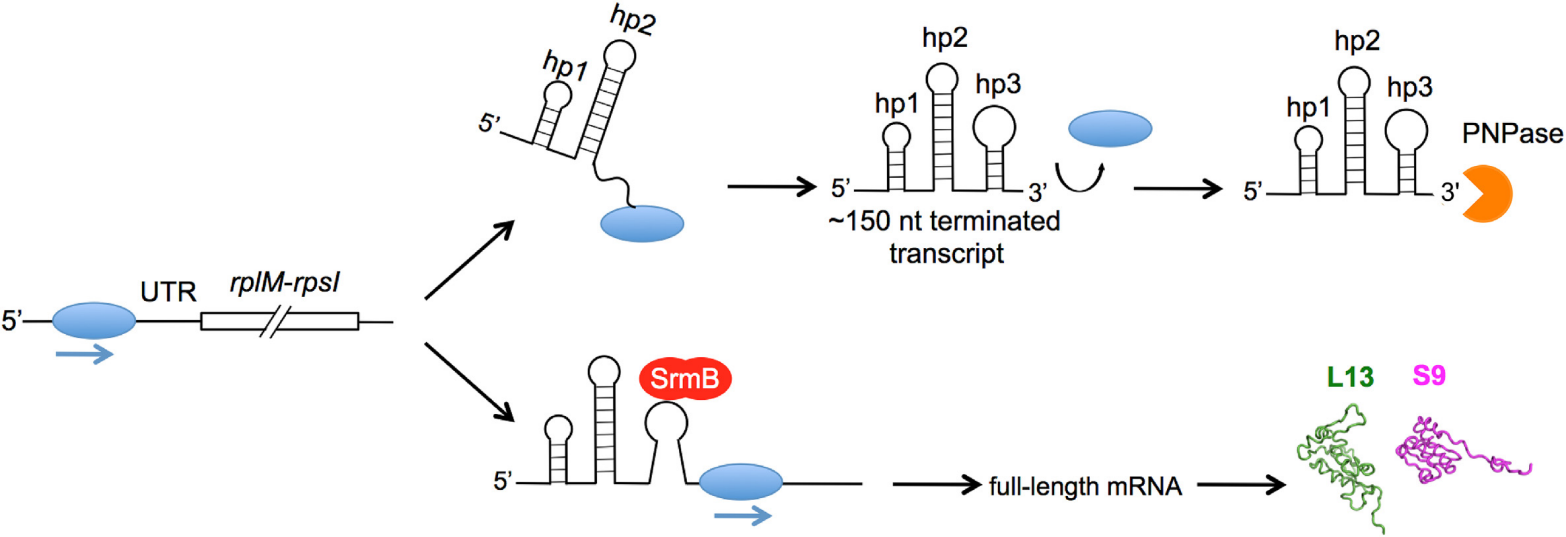
Model for SrmB dependent regulation of L13 and S9 synthesis. In this model, RNA polymerase (blue ovals) transcribes through the *rplM* 5′ UTR. A fraction of the RNA being transcribed is prematurely terminated downstream of hp3 and digested by PNPase (right, top). In the presence of SrmB (shown in red), hp3 is partially unwound, resulting in read-through through the 5′ UTR, and ultimately, increased L13 and S9 synthesis (right, bottom).

Based on the findings of this study, we propose a model for SrmB function in L13 and S9 synthesis (Figure 8). In this model, transcription is initiated at the *rplM-rpsI* promoter, and it either continues to yield the full-length *rplM-rpsI* transcript that is translated to produce L13 and S9, or is prematurely terminated within the 5′ UTR downstream of hp3. The proposed terminator contains a hairpin stem but lacks downstream poly(U) residues, a second characteristic of Rho-independent terminators, which might explain the substantial degree of read-through that is observed even in the absence of SrmB. When SrmB is present, we propose that it unwinds a part of the hp3 helical region that is external to the transcribing RNA polymerase, destabilizing hp3 and resulting in reduced termination. Consistent with the role of ATP as a cofactor for its helix-unwinding activity, mutations in the DEAD motif of SrmB reduced its ability to suppress termination *in vitro* and conferred a cold-sensitive growth phenotype *in vivo*. We also found that each of the five suppressors functions by reducing premature transcription termination (Figure 7), even though only one of these mutations lies in hp3, suggesting that elements external of hp3 also contribute to the efficacy of termination.

Previously, we have observed a reduced requirement of SrmB at higher growth temperatures (26), which, in light of our findings is also consistent with the model proposed, as SrmB-independent thermal unwinding at higher temperatures could play the same role as that of SrmB at lower temperatures to unwind secondary structures. Consistent with these expectations, prematurely terminated transcript levels were observed at lower levels when the strains were grown at higher temperatures, and additionally, the difference between wt and Δ*srmB* strains was also reduced (Figure 5D).

To the best of our knowledge, the mechanism by which SrmB functions on the *rplM-rpsI* transcript represents the first instance in which a DBP has been found to regulate gene expression by suppressing premature transcription termination. The previously described mechanisms through which DBPs were found to regulate gene expression in prokaryotes involve either translation regulation or mRNA stability, typically by remodeling secondary structures near the translation initiation site or those that determine RNA stability (39–41). That SrmB employs a different mechanism to regulate the *rplM-rpsI* transcript synthesis opens up the possibility that such a mode of regulation might be employed by other DBPs as well.

It is worth noting that prior work on this topic has suggested a direct role for SrmB in ribosome assembly. Thus, SrmB was shown to bind to pre-50S particles, and additionally, biochemical experiments showed that SrmB can form a complex with r-proteins L4 and L24 and their binding sites in 23S rRNA (12,15). Related to this observation, SrmB was originally identified as a multicopy suppressor of the *rplX19* mutation, which reduces the affinity of L24 for the ribosome (42), and *rplX19*Δ*srmB* strains are lethal (12). Together, these observations have suggested that L4, L24 and SrmB assist each other in binding to the nascent ribosome. Moreover, under conditions of rRNA over-expression, Δ*srmB* cells experience a strong growth and ribosomal defect even at 37°C, which can be suppressed by mutations in the 5S and 23S rRNAs (16). We propose that these previously observed defects of Δ*srmB* strains may not be due to reduced synthesis of L13 but to the absence of SrmB from the ribosome. Thus, SrmB likely has both direct and indirect roles in ribosome assembly, which we propose are not mutually exclusive, but act together to optimize ribosome assembly. This idea is consistent with the observation that a residual amount of 40S particles can still be observed in the ribosomal profiles of the *rplM* suppressor strains, as well from strains ectopically expressing L13 (Figure 2C and 4A, respectively). Nonetheless, since a major part of the ribosomal defects are alleviated by the suppressor mutations, it appears that the regulation of L13 and S9 synthesis represents the main mechanism through which SrmB regulates ribosome assembly.

A significant conclusion of this work is that ribosome assembly, a process that is most commonly associated with DBP function, is substantially regulated by SrmB through an indirect mechanism. To the best of our knowledge, indirect regulation of ribosome assembly has not been considered before, but in the light of our findings, we propose that SrmB does not represent an isolated case. Even in those examples where direct mechanisms have been proposed, it has not yet been demonstrated that an inability to perform the ribosomal rearrangement reactions ascribed to DBP function is actually responsible for the ribosomal defects that stem from DBP removal. Thus, it is possible that the real basis for the ribosomal function of such proteins is their regulation of factors that are important for ribosome assembly. Of note, ribosome assembly not only requires r-proteins, but also extra-ribosomal assembly factors that facilitate ribosomal assembly. Different organisms encode dozens to hundreds of such assembly factors (14,43), any of which could constitute the origin of ribosomal defects if they are regulated by DBPs. It will not be surprising, therefore, if future studies yield additional examples where the actual basis of DBP function in ribosome assembly is found to be not via direct action on the ribosome, but through the regulation of ribosomal or extra-ribosomal factors that are critical for ribosome assembly.
